# Effect of Aromatherapy Oil Applications on Strıae Gravidarum: A Systematic Review

**DOI:** 10.1111/srt.70354

**Published:** 2026-04-16

**Authors:** Tuba Güner Emül, Emine Kaplan Serin

**Affiliations:** ^1^ Department of Nursing, Obstetrics, Women's Health and Gynaecological, Diseases, Faculty of Nursing Mersin University Mersin Türkiye; ^2^ Department of Internal Medicine Nursing Mersin University, Faculty of Nursing Mersin Türkiye

**Keywords:** aromatherapy, pregnancy, randomized controlled trial, striae gravidarum

## Abstract

**Background:**

Striae gravidarum is a skin change that occurs during pregnancy, which does not pose a physiological threat to health but can cause psychological distress for individuals. Women may use moisturizers or aromatherapeutic oils in attempts to prevent the formation of striae.

**Aim:**

The purpose of this study is to review the literature on randomized controlled trials evaluating the effectiveness of aromatherapy oils in preventing striae gravidarum.

**Methods:**

The protocol for this systematic review and the writing of the article were based on the PRISMA‐P (Preferred Reporting Items for Systematic Review and Meta‐Analysis Protocols) criteria. The systematic review was conducted using the Google Scholar, PubMed, Web of Science, Scopus, and Cochrane Library databases between December 2024 and January 2025. Studies were selected by defining inclusion and exclusion criteria according to the PICOS method.

**Results:**

This systematic review included four randomized controlled trials with a total of participants. Two of the studies were published in 2024, one in 2022, and the other in 2020. This systematic review included four randomized controlled trials with a total of 671 pregnant participants. The essential oils used in the studies included coconut oil, rose oil, sesame oil, sweet almond oil, and olive oil. The studies found that coconut oil was effective in preventing striae gravidarum, while rose, sesame, and sweet almond oils were not effective.

**Conclusion:**

There is some evidence for the effect of aromatherapy in preventing striae gravidarum. However, larger sample sizes, higher‐quality studies, and randomized controlled trials with blinding methods are needed to evaluate the effectiveness of aromatherapy oils and determine their beneficial effects on pregnant women.

## 1 Introduction

Striae gravidarum (SG) is one of the most common skin changes occurring during pregnancy. It is observed in approximately 50%–90% of pregnancies and occurs with a higher incidence during the last trimester [1]. It is commonly noticeable on the breasts, abdomen, hips, thighs, and axillary regions [2]. Although the exact cause is not known, it is thought to develop due to a decrease in elastin and fibrillin levels that occurs during pregnancy. Family history, age, skin color, hormonal changes, amount of water consumed and weight gained during pregnancy are also known to be in the formation of stria gravidarum [3, 4]. SG, which does not completely disappear after birth, is seen as a cosmetic problem specific to women. These physiological changes that occur during pregnancy can affect the self‐esteem and body image of the pregnant woman. This situation may lead to the use of a number of applications (creams, lotions, aromatherapy oils, etc.) to attempt to prevent striae formation. When a study conducted in Türkiye was examined, it was determined that 57.5% of pregnant women used aromatherapy oils and these oils were olive oil, almond oil and St. John's wort oil. It was determined that 85.8% of these oils were used for striae that occurred during pregnancy [5, 6].

It has been reported that moisturizing the skin is effective in preventing SG, and creams, pomades and aromatherapy applications are used for this purpose, but studies to determine their effectiveness are limited [7, 8]. In a double‐blind randomized controlled trial (RCT) in Spain, a moisturizing cream containing rosehip oil was shown to reduce the severity of SG, prevent the occurrence of SG and halt the progression of existing SG [9]. In an experimental study conducted in Turkey, it was found that a 15 min massage with acibadem oil reduced the occurrence of SG. In contrast, an RCT conducted in Iran found that the use of olive oil and lanolin cream in the second trimester did not prevent the formation of SG [7]. In another study conducted in Iran, it was revealed that olive oil use in the second trimester was not effective in the occurrence of SG [10]. Canpolat et al. [7] found that 76% of women used topical moisturizers and emollients such as Vaseline, cocoa butter, and almond oil to prevent SG during pregnancy, but there was no relationship between the use of these substances and the development of SG. Aromatherapy applications applied in SG show us that there is still a need for well‐designed RCTs testing an oil or cream that prevents or reduces SG. As far as the literature was examined, it was determined that the number of studies on the prevention of striae in pregnancy was limited and there was no consensus in the studies. Therefore, it is thought that a systematic review of randomized controlled trials will make an important contribution to the literature.

## 2 Materials and Methods

The protocol for the systematic review and the writing of the article followed the PRISMA‐P (Preferred Reporting Items for Systematic Review and Meta‐Analysis Protocols) guidelines and was registered with PROSPERO (registration number: CRD42025630586). The literature search, selection of articles, and data extraction were independently reviewed by the authors. The quality of the studies was assessed independently by two researchers, and in case of disagreement, a third person was consulted.

### 2.1 Search Strategy

**FIGURE 1:**
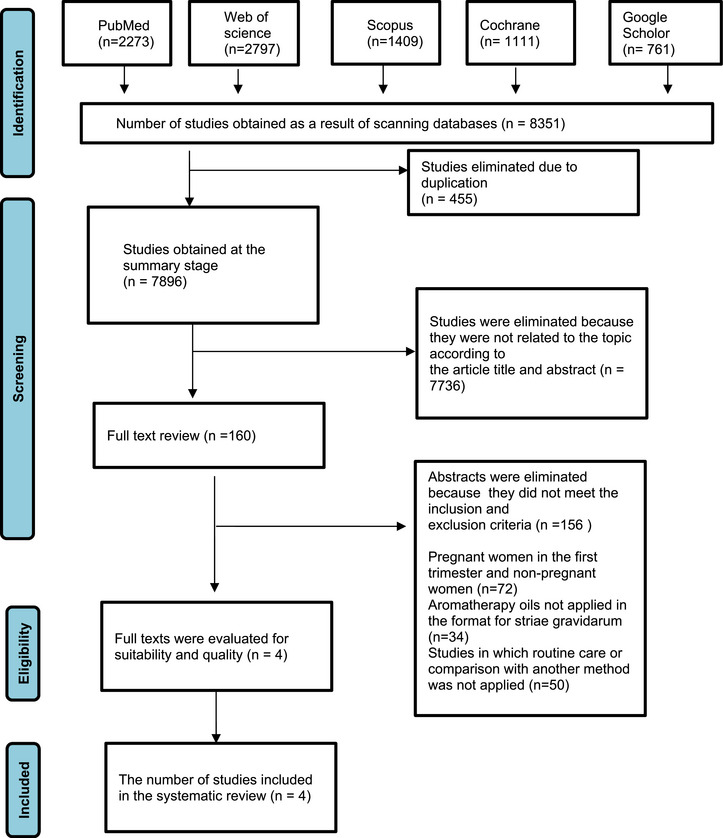
PRISMA flow chart of the screening process.

To identify studies on the effects of aromatherapy oils applied to stretch marks during pregnancy, a literature search was conducted using the Google Scholar, PubMed, Web of Science, Scopus, and Cochrane Library databases between November and December 2024. MeSH (Medical Subjects Headings) was used for the selection of English keywords. The search method was carried out using combinations of keywords and phrases such as “aromatherapy,” “striae distensae,” “randomized controlled trial” and “essential oil.” The number of studies was categorized according to the databases, and the PRISMA flow diagram shows the process of accessing the four studies included in the systematic review out of a total of 8351 studies (Figure 1).

### 2.2 Inclusion and Exclusion Criteria

The selection of studies included in this systematic review was determined according to the inclusion criteria created by the PICOS (P = Population, I = Implementation, C = Comparison group, O = Outcome, S = Study design) method. Inclusion and exclusion criteria are detailed in Table 1 [11].

**TABLE 1:** Inclusion and exclusion criteria in the study on the effect of aromatherapeutic oil applications on stria gravidarum.

	Inclusion criteria	Exclusion criteria
P	Pregnant women in the second and third trimesters	Pregnant women in the first trimester and non‐pregnant women
İ	Pregnant women who applied aromatherapy oils (in oil format) for striae gravidarum	Pregnant women who did not apply aromatherapy oils for striae gravidarum
**C**	Studies in which routine care or a different intervention was applied	Studies in which routine care or comparison with a different method was not applied.
**O**	Studies evaluating the outcomes related to striae in pregnancy	Studies that do not report outcomes related to striae in pregnancy
**S**	English experimental studies with accessible full text	Non‐experimental studies that are not in English and have inaccessible full text.

*Source*: Centre for Reviews and Dissemination [11].

### 2.3 Data Collection and Analysis

#### 2.3.1 Selection of Studies

In this systematic review, a total of 8351 studies were retrieved by searching databases based on the identified keywords. Duplicate articles were removed, and studies unrelated to the systematic review were excluded by reviewing titles and abstracts. The remaining articles were selected based on inclusion and exclusion criteria. If necessary, full‐text articles were examined to determine their eligibility. At the end of this process, four articles that met the inclusion criteria were found suitable for inclusion in the systematic review. Figure 1 presents the flowchart explaining the selection of studies according to the PRISMA guidelines.

#### 2.3.2 Data Extraction and Analysis

In the review, the titles and abstracts of the studies were initially read and evaluated, and then full‐text articles were examined in detail. The selections made by the researchers independently were compared, and a consensus was reached on differing opinions. A standard data extraction form was developed by the authors to summarize the data, and the data were evaluated accordingly. The data extraction form included information about the authors of the studies, the year of the study, the location of the study, the study design, sample size, the frequency of non‐pharmacological interventions, and the measurement tools used.

### 2.4 Assessment of Study Quality

In this systematic review, the methodological quality of the included studies was independently assessed by the first and second researchers. Any discrepancies between the evaluators were discussed, and consensus was reached.

The Joanna Briggs Institute (JBI) critical appraisal tool, revised by Barker and Stone and validated in Turkish by Nahcivan and Seçginli, was used to assess the methodological quality of all studies. This tool includes 13 items, with a total score ranging from 0 to 13. Each “yes” response was scored as 1 point, while “no,” “not specified,” or “not applicable” responses were scored as 0 points. Higher scores indicate higher methodological quality. In this review, the scores ranged from 10 to 12. For all randomized controlled trials, the Cochrane Risk of Bias 2 (ROB 2) tool was applied to evaluate the risk of bias across five domains: randomization process, deviations from intended interventions, missing outcome data, measurement of outcomes, and selection of reported results. The overall risk of bias for each study was classified as low, some concerns, or high. The quality of evidence for each outcome was assessed using the Grading of Recommendations, Assessment, Development, and Evaluation (GRADE) approach. This system evaluates certainty of evidence based on study limitations (risk of bias), inconsistency, indirectness, imprecision, and publication bias. Each outcome was rated as High, Moderate, Low, or Very Low. The results are presented in the Summary of Findings Table 2.

**TABLE 2:** Characteristics of studies examining the effects of aromatherapeutic oil applications on striae gravidarum.

Authors/ year of study/place of study	Study pattern	Aromatherapy oil used	Sample group and features	Intervention			Measurement tool	Main findings					ROB 2 Assesment	Quality of Evidence
				Duration/Amount	Frequency	Period								
**1**.Ulya et al., 2024/ Indonesia [12].	RKÇ	Virgin coconut oil (VCO)	Virgin Coconut oil group: 100 Average age: 26.4±4.3 Parity Primigravida: 38 Multigravida: 62 Gestational age: 20.4 ±3.2 Placebo group: 100 Average age: 26.6±4.1 Primigravida: 36 Multigravida: 64 Gestational age: 20.6 ±3.0	VCO Application Protocol: Dose: 5 mL (approximately 1 teaspoon) of A moisturizer cream without active ingredients was applied to the placebo group.	2 times/day	From the time of randomization at 32 weeks of pregnancy and until six weeks postpartum.	It has been evaluated using the Modified Striae Gravidarum Assessment Scale (SGAS)		**Virgin coconut oil (VCO)**	**Placebo**	**p**		Low risk	Moderate
								at 32 weeks of pregnancy	1.8±0,9	2.6±1.2	<0.001			
								six weeks postpartum	1.4±0.7	2.2 ±1.1	<0.001			
								VCO application has been significantly associated with lower striae gravidarum compared to placebo. Additionally, the VCO group showed a significant reduction in SG severity at the 32nd week and postpartum. No side effects were reported. As a result, topical VCO appears to be safe and effective in preventing new SG during pregnancy and reducing the severity of existing SG.						
**2**.Mirzeai et al., 2024/Iran [13].	RKÇ	Rose oil and sesame oil	Rose oil group: 50 Average age: 24.2±5.3 Nullipar gebe İkinci trimester Sesame group: 50 Average age: 25.8±5.0 Nullipar Second trimester Control group:50 Average age: 24.4±4.1 Nullipar second trimester	They received 1 g of intervention. (Each tube, 10% sesame oil and base cream, 10% Rosa damascena oil and base cream or control oil (base cream).	2 times/day	After 16 weeks of gestation 20 weeks without abdominal massage	Data collection involved the use of methodologies and complication checklists developed by Davey (Davey, 1972; Dhiman et al., 2009) and Kamini (Malakouti et al., 2017).	stria	rose	sesame	control	p	Some Concerns	Low
								none	%28	%26	%26	0.919		
								modarete	%18	%19	%20			
								severe	%28	%28	%27			
								There was no significant difference in baseline characteristics between the study groups. There was no effect on the incidence and severity of striae formation and associated itching in nulliparous pregnant women compared to the control group. Skin redness and itching were reported side effects following topical oil use.						
**3**.Kırca ve Gül, 2022/Turkey [14].	RKÇ	Olive oil	İntervention group:78 Average age: 29.1±4.5 Parity: Primigravida Third trimester Control group:78 Average age: 28±3.5 Parity: Primigravida Third trimester	10 cc (about 4 table spoons)	2 times/day	From 28th to 37th week of gestation without massage	Crack levels of the participants were evaluated using Fitzpatrick Skin Type Scale and Davey Severity Score, Fitzpatricks scoring.	Comparison of Davey's Severity Scores of the Groups					Low risk	Moderate
								Median (Min‐Max)	*p* Value					
								control	4 (0 ‐ 8)	<.001				
								intervention	2 (0 ‐ 8)					
								Fitzpatrick skin color scale						
									control	intervention	*p* Value			
								Tip 1	30.8	1.3	<.001			
								Tip 2	29.5	85.9				
								Tip 3	3.8	6.4				
								Tip 4	35.9	6.4				
								There was a statistically significant difference in the incidence and type of striae gravidarum between the intervention and control groups; 50% incidence and 85.9% type 2 striae were seen in the intervention group, while 69.2% incidence and 35.9% type 4 striae were seen in the control group.						
**4**.Sadat et al., 2020/Iran [15].	RKÇ	Almond and Sesame oil	Almond oil group:55 Average age: 28.01 Parity: primigravida Sesame oil group:55 Average age: 28.6 Parity:Primigravida: Control group:55 Average age: 31.4 Parity:Primigravida	1 mL. oil massaged into the skin of the abdomen for 5 min.	2 times/day	Between 16 and 36 weeks At week 36, women in each group were examined for subjective and objective criteria by three trained expert midwives unaware of the type of intervention.	Fitzpatrick Skin Type Scale and Davey Severity Score were assessed using Fitzpatricks scoring.	Frequency distribution of striae in groups of almond oil, sesame oil, and control at the end of the study					Some Concerns	Low
								Stria	Sesame oil	Almond oil	control	p		
								pozitif	33 (60.0)	35 (63.6)	32 (58.2)	0.837		
								negatif	22 (40.0)	20 (36.4)	23 (41.8)			
								It was determined that sesame and almond oil were not effective in the formation of striae gravidarum.						

### 2.5 Ethics Approval and Consent to Participate

This study is a systematic review based on literature screening and does not involve direct participation of humans or animals. Therefore, ethics committee approval was not required.

## 3 Results

The systematic review consists of four randomized controlled trials. In the review, the sample size ranged from 150 to 200, with a total of 671 pregnant participants. One of the studies included nulliparous women, two included primiparous women, and one included both primiparous and multiparous women. The studies were conducted in Iran (two studies), Indonesia (one study), and Turkey (one study). The evaluation tools used were the Modified Striae Gravidarum Assessment Scale (SGAS), participants' stretch mark levels using the Fitzpatrick Skin Type Scale and Davey Severity Score, Fitzpatrick scoring, the incidence and severity of striae on the abdominal skin using the Atwal numerical scoring system, and digital photography. Aromatherapy oils applied to pregnant women during the studies included coconut oil, rose oil, sesame oil, olive oil, and sweet almond oil (Table 2).

Primary Outcome, incidence and severity of SG, defined as the presence and extent of stretch marks on the abdominal, thigh, hip, and breast areas. Severity was assessed via SGAS, Fitzpatrick Skin Type Scale, Davey Severity Score, and Atwal scoring systems [12, 13, 14, 15]. Secondary Outcomes, side effects and maternal satisfaction, defined as reported skin reactions (redness, itching, allergic reactions) and women's subjective satisfaction with the oil application process [12, 13, 14, 15].

Ulya et al. [12] conducted a randomized controlled trial in Indonesia, involving pregnant women in the second trimester (14–27 weeks of gestation). The average age of the participants was 26.5 years, and the average gestational age was 20.5 weeks, including both primigravida and multigravida women. Inclusion criteria included being between 18 and 35 years old, having a singleton pregnancy, no previous history of SG, and no known allergies to coconut or its derivatives. The pregnant women were randomly assigned to receive either topical virgin coconut oil (VCO) or a placebo (standard emollient cream without active ingredients). Both VCO and the placebo were packaged in the same opaque containers to ensure blinding. Participants were instructed to apply about one teaspoon (5 mL) of the assigned product in a thin layer to their abdomen, breasts, thighs, and hips twice daily (morning and evening) from the time of randomization until six weeks postpartum. The SGAS, an approved tool for evaluating the severity of SG based on the number, length, width, color, and texture of the marks, was used in the study. No skin, allergic, or systemic side effects were reported. Women's satisfaction was high; participants reported that the oil was easy to apply, non‐greasy, and had a pleasant scent [12].

The application of VCO was significantly associated with a lower incidence of SG compared to placebo (25% vs. 45%, *p* < 0.05). Additionally, the VCO group showed a significant reduction in SG severity at the 32nd week and postpartum. This randomized controlled trial provides strong evidence that topical virgin coconut oil is a safe and effective intervention for preventing and reducing the severity of SG in pregnant women [12]. Mirzaei et al. [13] conducted a randomized controlled trial in Iran, involving 150 nulliparous women aged 18–35 years, between the 16th and 20th weeks of pregnancy. This was a triple‐blind study. The participants were randomly assigned in a 1:1:1 ratio to either the rose oil, sesame oil, or placebo group. For 20 weeks, the participants were instructed to apply 1 g (each tube contained 10% sesame oil with a base cream, 10% Rosa damascena oil with a base cream, and the control group received only the base cream) to their abdomen twice a day without massage. To assess striae and itching, the methods of Davey and Kamini were used as the data collection tools. Mild skin redness and itching were reported in a few participants, with no serious adverse events observed. Overall, women's satisfaction was moderate, as some participants expressed dissatisfaction due to minor skin irritation [13]. This study found that the use of 1 g of 10% rose oil and 10% sesame oil twice daily from the 16th to the 20th week of pregnancy until delivery did not have any effect on the incidence and severity of striae and associated itching compared to the control group in nulliparous women. For future studies, it is recommended to explore the use of these oils in different concentrations and different rose subtypes [13].

Kırca and Gül [14] conducted a study in Turkey with 156 pregnant women in their second trimester, all experiencing their first pregnancy. The average age of the participants was 28. Of the 156 women, 78 were in the intervention group, and 78 were in the control group. The participants' striae levels were evaluated using the Fitzpatrick Skin Type Scale, Davey Severity Score, and Fitzpatrick scoring system. Women in the intervention group were instructed to apply 10 cc (approximately 4 tablespoons) of extra virgin olive oil to their abdominal area twice daily (morning and evening) from the 28th to the 37th week of pregnancy, without massage. The intervention group participants were contacted by phone once a week by researchers to discuss their application status. No adverse effects were reported. Participants’ satisfaction was high, with weekly follow‐up and guidance contributing to increased adherence to the intervention [14]. The control group received no intervention. Women in the control group who used any other creams, oils, or medications were excluded from the analysis. The study found that in primigravidas, the use of olive oil did not prevent the formation of SG, but it was effective in reducing the severity compared to the control group. The researchers concluded that larger sample studies are needed to further investigate this topic [14].

Sadat et al. [15] conducted a randomized controlled study in Iran with 165 pregnant women in their second trimester. In the intervention group, women were instructed to apply a mixture of 1 mL sesame and almond oils, massaging it for 5 min, twice a day, starting from the 16th week until the end of the 36th week of pregnancy. The control group received no intervention. The Fitzpatrick survey, which classifies skin types, and Davey's striae scoring system were used for evaluation. No adverse effects were reported. Although women's satisfaction was not explicitly assessed, adherence to the intervention was high, likely due to regular follow‐up by the study staff [15]. The results of the study showed that sesame and almond oils were not effective in preventing SG. The researchers recommended conducting more studies to assess the effectiveness of sesame and almond oils [15].

## 4 Discussion

The studies included pregnant women with varying sample sizes and evaluation scales, indicating heterogeneity among the study populations. This review assessed the effects of essential oils on stretch marks. Participants were instructed to apply different oils as follows: 5 mL of coconut oil twice daily from randomization until six weeks postpartum [12]; 1 g of rose or sesame oil to the abdomen twice daily for 20 weeks without massage [13]; 10 cc of extra virgin olive oil twice daily to the abdomen until the 37th week [14]; and 1 mL of sesame and almond oil massaged for 5 min twice daily from the 16th to 36th week [15]. Control groups varied: one received a placebo cream without active ingredients [12], another applied a base cream [13], and in the remaining studies no intervention was provided [14, 15]. Aromatherapy is a therapeutic method aimed at preserving and enhancing physical and mental health, using essential and carrier oils obtained from the flowers, leaves, fruits, roots, and stems of plants [16]. During pregnancy, aromatherapy is used to promote relaxation, alleviate fatigue, and reduce physical symptoms. Only oils approved for use during pregnancy should be utilized in small doses after the second trimester. Oils considered safe for pregnant women include cardamom, chamomile, frankincense, geranium, mandarin, lemon, lemon balm, lavender, ylang‐ylang, jasmine, sandalwood, rose, tea tree, rosemary, cedarwood, eucalyptus, bergamot, patchouli, bitter orange, cypress, and orange blossom oils [1, 17, 18, 19, 20, 21]. Aromatherapy during pregnancy is used to promote relaxation, alleviate fatigue, and reduce physical symptoms. In the application of aromatherapy during pregnancy, essential oils should be used in small doses after the second trimester, and only oils that are approved for use during pregnancy should be utilized. The oils considered safe for use by pregnant women include cardamom, chamomile, frankincense, geranium, mandarin, lemon, lemon balm, lavender, ylang‐ylang, jasmine, sandalwood, rose, tea tree, rosemary, cedarwood, eucalyptus, bergamot, patchouli, bitter orange, cypress, and orange blossom oils [1, 17, 18, 19, 20, 21]. Akbulut and Bolsoy [5] found in their study that 42.5% of pregnant women used herbal oils and aromatherapy oils. The primary reason for using these oils among pregnant women was to prevent SG (85.8%). The most preferred oils were olive oil (49%) and almond oil (26.5%) [5]. A review of the literature reveals that studies on the use of aromatherapy oils to prevent SG are limited in number [12, 13, 14, 15, 22].

Coconut oil and its components have been found to show low allergenicity and immune response when applied topically to the skin, suggesting that it could be beneficial for individuals with sensitive or reactive skin [23]. When reviewing studies on coconut oil, it has been found to support skin health by improving elasticity, hydration, and barrier function [24]. Olive oil can be used on its own or as a carrier oil due to its medicinal properties. Recent studies have shown that the benefits of olive oil for the skin are not only derived from its fatty acids but also from the tocopherols, phytosterols, phospholipids, and squalene it contains. In one study, the external application of olive oil was found to increase the hydration of the stratum corneum in 150 healthy participants. Olive oil is commonly used to prevent skin cracks [8]. Like olive oil, almond oil also contains a high amount of oleic acid and has a moderate comedogenic character. It is suitable for dry and sensitive skin and has been found to reduce moisture loss. In one study, olive oil was found to be effective in preventing the formation of SG and reducing the associated itching [22].

A study found that a 15 min massage with almond oil, starting from the 19th week of pregnancy, reduced the development of SG. The massage with almond oil was found to be effective in preventing the formation of SG in the abdominal area. However, the application of bitter almond oil with the same frequency had no effect on the development of SG. Planning more studies investigating the effects of massage applied at different pregnancy weeks and durations on SG development could provide more insights on this matter [25]. In studies evaluating the effects of aromatherapy oil applications on SG, it was found that coconut oil is effective in reducing SG, while olive oil does not prevent SG formation but is effective in reducing its severity. In contrast, rose, sesame, and almond oils were found to have no effect on SG formation. Researchers have collectively suggested that more randomized controlled trials should be conducted to further investigate these effects.

## 5 Limitations

This systematic review has several limitations. First, descriptive studies were not included. Second, only studies published in English were considered. Additionally, a meta‐analysis could not be conducted due to heterogeneity among the included studies regarding sample sizes, intervention protocols, and outcome assessment methods.

## 6 Conclusion

This systematic review indicates that certain aromatherapeutic oils, particularly coconut oil, may be beneficial in preventing SG and reducing their severity in pregnant women. Olive oil may help reduce severity but does not prevent formation, while rose, sesame, and sweet almond oils appear ineffective. Minimal side effects were reported, and women generally reported moderate to high satisfaction with the interventions. To provide stronger clinical recommendations, further large‐scale, well‐designed randomized controlled trials are necessary to assess efficacy, optimal dosing, and long‐term safety of aromatherapeutic oils during pregnancy.

## Funding

The authors have nothing to report.

## Conflicts of Interest

The authors declare no conflicts of interest.

## Supporting information




**Supplementary Table 1**. Search Strategy for Each Database. **Supplementary Table 2**. Excluded Studies and Reasons for Exclusion

## Data Availability

All data generated or analyzed during this study are included in this published article and its Supporting Information.
