# Supplementary material for: Effect of Aromatherapy Oil Applications on Strıae Gravidarum: A Systematic Review
Source: Skin Res Technol. 2026 Apr 16;32(4):e70354. doi: 10.1111/srt.70354 (PMC13084530; doi:10.1111/srt.70354)
Supplement: Supplementary file 1 — Supplementary Table 1. Search Strategy for Each Database. Supplementary Table 2. Excluded Studies and Reasons for Exclusion [file SRT-32-e70354-s001.docx]

**Supplementary Table 1. Search Strategy for Each Database**

| **Database** | **Search Date** | **Search Terms / Keywords** | **Filters / Limits Applied** |
| --- | --- | --- | --- |
| PubMed | 01-Jan-2025 | ("Striae Gravidarum" OR "stretch marks") AND ("aromatherapy" OR "essential oil) AND randomized controlled trial | English, Humans, RCT years (2020-2025), Full text not available |
| Scopus | 01-Jan-2025 | ("Striae Gravidarum" AND ("aromatherapy" OR "essential oil"))AND randomized controlled trial | English, Articles years (2020-2025) Full text not available |
| Web of Science | 01-Jan-2025 | TS=("Striae Gravidarum" OR "stretch marks") AND TS=("aromatherapy" OR "essential oil")AND randomized controlled trial | English, Humans, Full text not available  years (2020-2025) |
| Cochrane Library | 01-Jan-2025 | ("Striae Gravidarum" OR "stretch marks") AND ("aromatherapy" OR "essential oil") AND randomized controlled trial | English, RCTs, Full text not available  years (2020-2025)  English, RCTs,  years (2020-2025) |

Google scholor 01-Jan-2025 ("Striae Gravidarum" OR "stretch marks") AND ("aromatherapy" OR "essential oil") AND randomized controlled trial

**Supplementary Table 2. Excluded Studies and Reasons for Exclusion**

| **Excluded Studies and Reasons** | Number of Studies |
| --- | --- |
| Pregnant women in the first trimester and non-pregnant women | 72 |
| Aromatherapy oils not applied in the format for striae gravidarum | 34 |
| Studies in which routine care or comparison with another method was not applied | 50 |
|  |  |
